# Eligibility for antiamyloid treatment: preparing for disease-modifying therapies for Alzheimer’s disease

**DOI:** 10.1136/jnnp-2024-333468

**Published:** 2024-06-11

**Authors:** Ruth Dobson, Katherine Patterson, Reshad Malik, Uttara Mandal, Hina Asif, Ros Humphreys, Michael Payne, Eng O-Charoenrat, Lauren Huzzey, Adam Clare, Kate Green, Maija Morton, Catrin Sohrabi, Navreen Singh, Amirtha Pasupathy, Milan Patel, Sam Whiteman, Kate Maxmin, Nicholas Bass, Bhavya Gupta, Claudia Cooper, Charles Marshall, Rimona Sharon Weil, Catherine J Mummery

**Affiliations:** 1 Centre for Preventive Neurology, Wolfson Institute of Population Health, Queen Mary University of London, London, UK; 2 Academic Health Science Centre, UCL Partners, London, UK; 3 National Hospital for Neurology and Neurosurgery, London, UK; 4 Haringey Memory Service, Barnet Enfield and Haringey Mental Health NHS Trust, London, UK; 5 Enfield Memory Service, Barnet Enfield and Haringey Mental Health NHS Trust, London, UK; 6 Barnet Memory Service, Barnet Enfield and Haringey Mental Health NHS Trust, London, UK; 7 Camden Memory Service, Camden and Islington Mental Health and Social Care Trust, London, UK; 8 Tower Hamlets Memory Service, East London NHS Foundation Trust, London, UK; 9 East London NHS Foundation Trust, London, UK; 10 Centre for Psychiatry, Queen Mary University of London, London, UK; 11 Dementia Research Centre, University College London, London, UK; 12 Department of Neurodegenerative Disease, National Hospital for Neurology and Neurosurgery, London, UK

**Keywords:** memory, dementia, alzheimer's disease, clinical neurology

## Abstract

**Background:**

Disease-modifying therapies (DMTs) for Alzheimer’s disease (AD) have early evidence of efficacy. Widespread delivery of DMTs will require major service reconfiguration. Treatment pathways will need to include triaging for eligibility, regular infusions and baseline and follow-up MRI scanning. A critical step in planning is provision of real-world estimates of patients likely to be eligible for triaging, but these are challenging to obtain.

**Methods:**

We performed a retrospective service evaluation of patients attending five memory services across North and East London and a national specialist cognitive disorders service. We examined the likely proportion of patients who would (1) be referred for triaging for DMTs and (2) potentially be suitable for treatments.

**Results:**

Data from a total of 1017 patients were included, 517 of whom were seen in community memory services and 500 in a specialist clinic. In the memory services, 367/517 (71%) were diagnosed with possible AD. After exclusions of those in whom cognitive and frailty scores, MRI contraindications or anticoagulant use indicated they would be unlikely to be suitable, an estimated 32% would be eligible for triaging. In the specialist cognitive clinic, where additional investigations are available, 14% of those seen (70/500) would be potentially eligible for treatment.

**Conclusions:**

While a sizeable proportion of patients attending memory clinics may be referred for triaging for DMTs for AD, only a minority are likely to be suitable for these, as demonstrated in patients seen in specialist cognitive services. This will need to be considered when designing pathways for DMT delivery.

**WHAT IS ALREADY KNOWN ON THIS TOPIC**

- Alzheimer’s disease is the most common cause of dementia.
- New treatments with the potential to modify the disease course are currently going through approval processes in the UK and the rest of the world.
- In order to plan delivery of these new treatments, real-world estimates of the numbers of patients likely to be suitable are urgently needed.

**WHAT THIS STUDY ADDS**

- This study examined 1017 people who attended either memory services (517 people) or a specialist cognitive service (500 people).
- It took into account the diagnoses that were made and looked at detailed information about how severely affected people were by memory changes and frailty, as well as reasons for exclusions for new treatments.
- The study showed that a high proportion of people (32%) attending memory services are likely to be referred for consideration of new disease-modifying therapies (DMTs).
- Only 14% of people attending specialist cognitive services, where amyloid biomarker tests were available, were potentially eligible for these new treatments.
- <1% of patients attending community memory clinics had amyloid biomarker testing performed, making this an urgent area of need for service development to enable identification of suitable patients for DMT.

**HOW THIS STUDY MIGHT AFFECT RESEARCH, PRACTICE OR POLICY**

- This study provides best possible estimates of the proportion of people likely to be referred for new DMTs in AD. It will help in planning how these new treatments will be delivered in clinical settings.

## Introduction

Alzheimer’s disease (AD) is the most common cause of dementia. Approximately 944 000 people in the UK live with dementia, 60%–80% of whom have AD.^1 2^ Until recently, treatment has been limited to cholinesterase inhibitors and memantine, which provide some symptomatic benefits, alongside services to support people with AD and their families. Recent clinical trials of monoclonal antibodies to remove beta-amyloid have shown early evidence of efficacy against both amyloid-related biomarkers and clinically detectable outcomes. Aducanumab (now discontinued) was the first to report efficacy in slowing clinical deterioration in AD,^3^ prompting the US Food and Drug Administration (FDA) accelerated approval for use in dementia of Alzheimer’s type.^4^ In July 2023, lecanemab^5^ received traditional approval from the FDA,^6^ followed within weeks by reports that donanemab slowed cognitive deterioration in patients with early AD.^7^ In the UK, lecanemab and donanemab have both been granted ‘breakthrough therapy’ designation, a likely precursor to licensing, with approvals expected from the Medicines and Healthcare products Regulatory Agency (MHRA) and the National Institute for Health and Care Excellence (NICE) by mid-2024. Disease-modifying therapies (DMTs) are thus highly likely to be a reality for UK patients with AD in the next few years.

It is clear that delivering these treatments will require major service reconfigurations with a focus on early, robust, pathology-specific diagnosis of disease-causing cognitive symptoms.^8^ Multidisciplinary involvement will be essential, alongside access to fluid biomarker laboratories or Positron Emission Tomography (PET) imaging for confirmation of beta-amyloid status. Infusion nurses will need to support at least monthly infusions. Neuroradiologists will be needed to enable accurate diagnosis and ensure appropriate eligibility criteria are met on MRI, followed by a need for access to and interpretation of regular MRI scans to monitor for changes including amyloid-related imaging abnormalities (ARIA); those who start treatment will require four to five MRI scans over the first year of treatment.^8^ Clinicians will need to support people to make informed decisions around treatments, especially those with coexisting multimorbidity.

With few exceptions, dementia care in the UK is currently centred around psychiatry-led memory clinics in the community. The potential roll-out of DMTs presents a major challenge in terms of service delivery and has real potential to amplify existing inequities in service access. It is overwhelmingly likely that these treatments will not be administered in existing community memory services.

A critical first step in setting up treatment pathways is estimating the number of patients likely to be eligible for anti-amyloid therapies. AD is the most common cause of dementia, so the potential pool is extremely large. However, clinical trial evidence for efficacy thus far is only at the milder stages, for patients with Mini-Mental State Examination (MMSE) Scores at or above 20/30.^3^ Given the physiological demands of frequent infusions and potential for side effects from infusion reactions and ARIA, physical frailty will need to be considered in discussions regarding eligibility, as well as presence of anticoagulants or bleeding disorders. While frailty in itself was not an exclusion criterion from clinical trials, it is a proxy measure for ability to tolerate high-intensity therapy across medical diagnoses, and so forms part of appropriate use recommendations (AURs).^8^ Other exclusions are likely to be inability to undergo MRI scanning due to requirements for MRI monitoring, and the need to screen for imaging abnormalities including cerebral microbleeds, cerebral amyloid angiopathy and significant cerebral infarctions or brain injuries. Screening for *APOE*4 alleles is a further consideration, as homozygotes are more likely to develop ARIA, making homozygosity a potential absolute contraindication to treatment.^8 9^

Pathways for DMTs are likely to involve several stages: a referral for triaging from wider memory services, followed by mechanisms to select patients eligible for DMTs in specialist services, using more specialised testing for presence of beta-amyloid with either PET imaging or fluid measures. Being able to estimate the number of patients at each of these stages is critically needed to inform planning, design and delivery of these new treatments. Dementia subtype is not reliably available from population data or even hospital records.^10^ Even more challenging to obtain at population level are estimates of cognitive and functional severity, which will be early and key elements of triage. A potential eligibility gap according to social determinants of health remains a real concern; people from minority ethnic backgrounds, those with caring responsibilities and those with lower educational attainment may seek care later and hence miss a window of opportunity for access.

We therefore aimed to provide a best possible estimate of the proportion of patients likely to be eligible for triage into DMT services. We sought to examine patients in both local memory clinics and in a specialist cognitive service. We involved clinics affiliated with University College London Partners (UCLP), a network of clinical services encompassing a diverse population of 5 million people. We retrospectively examined the proportion of patients who received a diagnosis of AD, and of those, the proportion potentially suitable for DMT. This enables an estimation of the likely demand for referral to dementia DMT services, facilitating service planning and development across the National Health Service (NHS).

## Methods

### Populations

#### Memory clinics

Memory clinics (referred to as sites) across North and East London within the UCLP region were approached. Five sites responded and provided fully anonymised data for inclusion. Each site was asked to provide data on 100 consecutive and otherwise unselected patients seen in memory clinics within a 6-month period in 2022 via retrospective case-note review.

#### Specialist cognitive clinic

A retrospective case-note review was performed at a specialist cognitive disorders service at the National Hospital for Neurology and Neurosurgery, London, between November 2021 and May 2022. Criteria for referral to the cognitive disorders clinic are being under the age of 65 or having a complex, rapid or atypical form of dementia, or chronic neurological or psychiatric disease with progressive cognitive deficit. 500 consecutive patients seen in five clinics within the service were included.

### Data collection

A dedicated data capture form with restricted fields was used by each site to ensure data validity. Inclusion criteria were all patients assessed within the Memory Clinic or Cognitive Service within the given timeframe. Retrospective data were collated at each site and fully anonymised at the point of collection. No identifiable patient data were used in any analysis.

We collected demographic information including age, sex, diagnosis and ethnicity categorised according to Office for National Statistics high-level terms and date of assessment in the clinic. Ethnicity data were not available for patients attending the specialist cognitive disorders service. Where available, native language was recorded from clinical records and assumed to be English if not specified. In the specialist cognitive disorders service, source of referral was also collected.

Severity of cognitive impairment was assessed using either Addenbrooke’s Cognitive Examination (ACE), Rowland Universal Dementia Assessment Scale (RUDAS) or MMSE as recorded in clinical notes. Where MMSE Score was available, this was preferentially used. Where not available, RUDAS and ACE Scores were used. A Rockwood Clinical Frailty Score was either captured directly if recorded, or carried out based on available information in the clinical record^11^; in the specialist cognitive clinic, these were applied by a geriatrician experienced in frailty. Use of anticoagulant therapy was noted where available. We also collected information on whether the patient had undergone MRI scanning and use of fluid biomarkers.

### MRI exclusions

In order to evaluate potential exclusions based on MRI findings, data from two sites with complete MRI reports for all patients were evaluated. Two raters (RSW and RD) rated MRI reports independently; where these diverged, they were resolved by discussion. MRI reports were examined in 475/500 patients seen at the specialist cognitive clinic by a single rater (KP) with AUR exclusion criteria applied.

### Triage and eligibility evaluation

As populations seen in community memory services and specialist cognitive services represent distinct groups, with potential for different diagnostic rates resulting from different referral patterns, clinical assessments and access to investigations, they were examined separately. Given that DMTs are not currently available for dementia, we did not presuppose whether rates of AD or potential DMT eligibility would be higher in one group or the other.

Given that likely pathways will flow from memory clinics via a triage route, and subsequently into cognitive services we defined two eligible groups:

1. Those eligible for triaging from the memory clinic populations.
2. Those likely to be eligible for treatment from the cognitive clinic population.

As eligibility criteria for future NHS roll-out of DMTs are not yet known, we used expert consensus to define likely eligibility. We judged that patients meeting the following criteria were likely to be eligible:

1. Diagnosis:
  - For patients assessed in the memory clinic sites, patients were deemed potentially suitable for triaging if they had a diagnosis of AD, mixed dementia, dementia not specified, suspected dementia, mild cognitive impairment, awaiting diagnosis and other (where no specific diagnosis was given that would be a clear exclusion for DMT). These wide criteria were used on the basis that fluid biomarker or PET confirmation of AD is not usually available in memory clinics; we wanted to account for diagnostic variability and understand the potential maximum impact on triage services that may be required to refine diagnoses. People with the following diagnoses were judged likely unsuitable for onward referral for DMTs: alcohol-related dementia, mood disorder, vascular dementia, Parkinson’s dementia, dementia with Lewy bodies, non-organic or functional dementia, or memory disturbance related to underlying psychiatric disorder.
  - In the specialist cognitive service, a diagnosis of AD was considered where this had been positively determined based on clinical history, clinical examination and supported by imaging, determining eligibility for treatment. We further noted confirmatory biomarker evidence (usually cerebrospinal fluid (CSF)) and/or amyloid PET.
2. Degree of cognitive impairment: On the basis that those with higher cognitive scores are likely to be eligible for DMTs, we used an MMSE cut-off score of ≥20 in both cohorts, as per the landmark TRAILBLAZER-ALZ2 study.^12^ Where a RUDAS was performed, we used a RUDAS Score of 20 as equivalent to MMSE=20.^13^ Although the MMSE is captured with the ACE, it was often not recorded, and an ACE Score of 53 was used as equivalent to MMSE=20.^14^ Where MMSE or cognitive scoring was missing, we used a frailty score ≥5 as an indicator of likely MMSE ≥20.
3. Frailty: Rockwood Clinical Frailty Score ≤5 was used to define eligibility for either triaging or treatment on grounds of including only mild frailty.^11^
4. Anticoagulation: Use of anticoagulant therapy (including use of warfarin, novel oral anticoagulants and directly acting oral anticoagulants) was considered a likely exclusion for both triaging and treatment, based on AUR.^15^
5. MRI: We used AUR^15^ to determine MRI exclusions. Exclusion criteria were as follows: >4 microhaemorrhages, >2 lacunar infarcts or infarct in major vascular territory, severe subcortical hyperintensities (Fazekas 3), MRI evidence of non-AD dementia, recent stroke and major intracranial pathology potentially causing cognitive impairment.

### Statistical analysis

Demographic data were reported across the whole population, and within each site. We did not evaluate proportions of available assessments at site level, to prevent unintended assessments of service quality using imperfect proxies. Standard descriptive statistics were used. For comparisons between groups, we used χ^2^ to compare proportions and analysis of variance to compare normally distributed data across multiple groups with a significance threshold of p<0.05.

## Results

### Patient populations

#### Memory clinics

Five memory clinics from North and East London (Barnet, Camden, Chase Farm, Haringey and Tower Hamlets Memory Services) contributed anonymised clinical information to this service evaluation, providing data on a total of 542 individuals. Complete core data (age, gender and diagnosis including unknown/other) were available for 517 individuals, forming the cohort included in the final analysis. The majority of these patients represented new referrals to services, as longitudinal follow-up in community memory service clinics is unusual.

Clinical reviews occurred between January and June 2022. There were slightly fewer males than females across the entire cohort (40.6% male). Mean age was 79.4 years (SD=8.8), with 72 (13.9%) aged under 70. Site 5 reported a significantly younger population than all other sites (p<0.00001); however, some services exclusively review older patients. Ethnic distribution differed substantially between sites, ranging from 39% to 71% White patients (p<0.00001, χ^2^). Full demographic characteristics are given in table 1. Given variation in demographic characteristics and diagnostic codes, we did not explore the impact of these on eligibility.

**Table 1:** Demographic details of patients seen at five memory clinics across North and East London and in the specialist cognitive service at the National Hospital for Neurology and Neurosurgery

	Entire memory clinic cohort	Site 1	Site 2	Site 3	Site 4	Site 5	Specialist cognitive service
Number	517	100	100	100	127	89	500
M:F; %M	210:307 (41%)	41:59 (41%)	37:63 (37%)	42:58 (42%)	51:76 (40%)	38:51 (43%)	265:235 (53%)
Age (years), mean (SD)	79.4 (8.8)	81.2 (7.1)	80.2 (8.5)	79.9 (7.8)	81.7 (7.0)	72.2 (11.9)	66.2 (12.13)
Proportion aged <70 years, n (%)	72 (14%)	9 (9%)	12 (12%)	12 (12%)	4 (3%)	35 (39%)	290 (58%)
Ethnicity, n (%)							NA
White	302 (58%)	53 (53%)	69 (69%)	54 (54%)	90 (71%)	35 (39%)	
Black	32 (6%)	10 (10%)	7 (7%)	9 (9%)	4 (3%)	2 (2%)	
Asian	103 (20%)	21 (21%)	8 (8%)	18 (18%)	19 (15%)	38 (43%)	
Other/mixed	54 (10%)	15 (15%)	12 (12%)	16 (16%)	7 (6%)	4 (5%)	
Unknown	26 (5%)	1 (1%)	4 (4%)	3 (3%)	7 (6%)	11 (12%)	
English as a foreign language, n (%)	79 (25%) (available for 323)	25 (25%)	NA	29 (29%)	25 (20%)	NA	

NA, not available.

#### Specialist cognitive service

Data from 500 consecutive patients seen across five neurology-led cognitive clinics were included. These comprised four clinics for general cognitive disorders and one for general cognitive disorders, with some subspeciality Lewy body dementia.

This cohort comprised 144 new patients and 356 follow-up visits. In contrast to the memory clinics, there were slightly fewer females (47% females, 53% males). The population was substantially younger than those seen in community clinics, with a mean age of 66.2 years (SD=12.13). 290 (58%) were aged under 70. The most frequent diagnosis was AD (177, 35.4%), followed by frontotemporal dementia (72, 14.4%). Demographic characteristics are given in table 1.

### Eligibility for triage and potential treatment with DMTs for AD

Despite the variation in age and ethnic background of the populations seen in each clinic, the proportion of patients potentially suitable for triaging for DMTs was remarkably consistent across community services. Across the cohort, 70.9% had a diagnosis of AD, mixed disease or non-specified dementia, which could warrant referral for triaging for DMT (68%–76% across sites; table 2). Cognitive assessment was documented within 18 months of assessment in 93.2% of cases, and the overall proportion of patients with MMSE ≥20 or equivalent was 76.8% (71%–82% across sites; table 2). Estimates of frailty revealed low levels of frailty, with 74.5% scoring ≤5, indicating only mild frailty, on the Rockwood Frailty Score (69%–83% across sites).

**Table 2:** Factors relating to DMT eligibility in memory clinics in North and East London and the specialist cognitive service at the National Hospital for Neurology and Neurosurgery

	Entire memory clinic cohort (n=517)	Site 1 (n=100)	Site 2 (n=100)	Site 3 (n=100)	Site 4 (n=126)	Site 5 (n=89)	Specialist cognitive service (n=500; 177 AD included in analysis)
Dementia/AD diagnosis potentially eligible for DMT	367 (71%)	73 (73%)	68 (68%)	76 (76%)	88 (70%)	62 (70%)	177
Formal cognitive assessment documented within 18 months	482 (93%)*	–	–	–	–	–	156 (88%)
MMSE ≥20*	397 (77%)	71 (71%)	82 (82%)	75 (75%)	101 (80%)	68 (76%)	74 (47%)
Rockwood frailty score ≤5†	320 (75%†)	69 (69%)	70 (70%)	76 (76%)	105 (83%)	NA	107 (60%)
Potentially eligible for DMT¶	276 (53%)	41 (41%)	42 (42%)	61 (61%)	76 (60%)	56 (63%)	68 (38%)
Neuroimaging performed, n (%)‡	388 (75%)	95 (95%)	50 (50%)	70 (70%)	85 (68%)	88 (99%)	177 (100%)
MRI results available, n (%)	239 (46%)	73 (73%)	23 (23%)	55 (55%)	29 (23%)	59 (66%)	174 (98%)
Amyloid biomarkers assessed (plasma/CSF/amyloid-PET), n (%)	2‡ (0.4%)	0 (0%)	NA	NA	NA	2‡ (2%)	109§ (62%)
FDG-PET	NA	NA	NA	NA	NA	NA	4 (2%)

*Or equivalent ACE/RUDAS.

†Data not available for site 5 so excluded from all proportions.

‡Does not capture where imaging performed from primary care prior to referral.

‡In a small proportion of memory clinics, plasma p-tau was assessed.

§In the specialist cognitive clinic, CSF or amyloid-PET was assessed.

¶Calculated as those with potentially eligible diagnosis, MMSE ≥20 and Rockwood ≤5. Where Rockwood missing for site 5, patients were included if they had a potentially eligible diagnosis and MMSE ≥20. Those patients with only MMSE available (i.e. those without Rockwood) were excluded from the cohort described in figure 2.

ACE, Addenbrooke’s Cognitive Examination; AD, Alzheimer’s disease; CSF, cerebrospinal fluid; DMT, disease-modifying therapy; FDG-PET, fluorodeoxyglucose positron emission tomography; MMSE, Mini-Mental State Examination; PET, positron emission tomography; RUDAS, Rowland Universal Dementia Assessment Scale.

In the specialist cognitive service, cognitive testing results were available in 156/177 patients with AD within 18 months of the audit. Limitations in available data were related to ongoing remote reviews following the COVID-19 restrictions. 47% (74/156) of those patients had a documented MMSE ≥20. Frailty levels were slightly higher than in the memory clinics, with 60% (107/177) having a frailty score of ≤5 (table 2).

### Imaging and paraclinical investigations

#### Memory services

388/517 (75%) patients seen in community memory services had undergone brain imaging (CT and/or MRI). Of those potentially suitable for triaging for DMT (table 2), 161 had undergone neuroimaging with results available in memory clinics, of whom 98 had MRI results available, suggesting they could have further MRI scanning. Evaluation of MRI results with reference to contraindications to DMTs was performed for two sites with available MRI results. In the memory services cohort, only two patients had fluid biomarker evaluation.

#### Specialist cognitive services

492/500 patients had undergone CT or MR imaging with results available for review. The majority had MRI (475), with 17 patients having undergone CT due to MRI contraindications. Other investigations included fluorodeoxyglucose-PET (FDG-PET) in 4.6%, amyloid-PET in 6.6% and CSF analysis in 31% (table 2). Of those with an AD diagnosis, use of extended biomarkers was higher with 62% (109/177) having biomarker confirmation of amyloid pathology, either via CSF or amyloid-PET, (table 2).

A subgroup analysis of patients with AD (n=177) revealed a relatively high proportion with rare variants. These were 32% young onset AD, 11% posterior cortical atrophy; 11% primary progressive aphasia, 9% familial AD and 2% corticobasal syndrome.

#### Eligibility for DMTs: memory services

**Figure 1:**
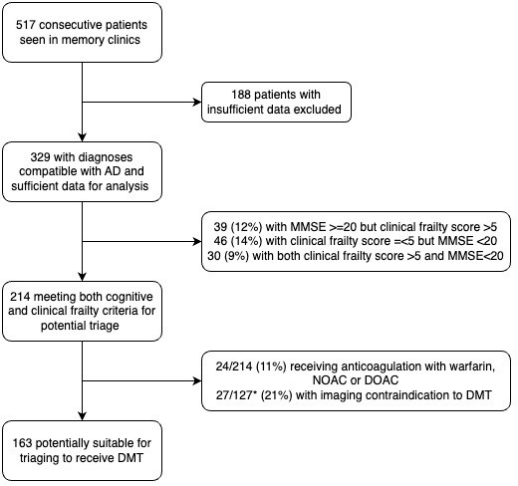
Memory clinic population potentially suitable for triaging for DMT. Proportion likely to be suitable for referral for triaging is highlighted, based on lower cognitive severity and clinical frailty, MRI contraindications and anticoagulant use. ^+^329 included where sufficient clinical data were available for review; those without both MMSE and Rockwood available were excluded *Imaging was reviewed in 127 cases. AD, Alzheimer’s disease; DMT, disease-modifying therapy; DOAC, directly acting oral anticoagulant; MMSE, Mini-Mental State Examination; NOAC, novel oral anticoagulant.

Of 329 patients seen in community memory clinics with AD-compatible diagnoses and sufficient clinical data to make an assesment, 214 (65%) had cognitive severity and clinical frailty at levels suitable for referral for DMT (figure 1). 24 were taking anticoagulation, and therefore not eligible for DMT. Imaging reports were available for 127 patients from two sites. Of these, 27 patients were not suitable for DMT triaging, based on MRI findings according to AURs.^15^ This left a total of 163 patients (31.5% of all patients seen) suitable for triaging for DMTs (figure 1). In the memory services cohort, two patients had fluid biomarkers assayed. Neither were suitable for DMT on the basis of a diagnosis other than AD.

#### Eligibility for DMTs: specialist cognitive service

**Figure 2:**
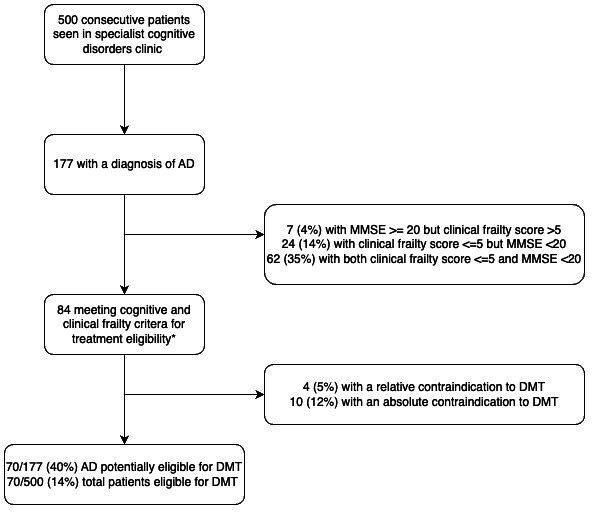
Specialist cognitive clinic population potentially suitable for DMT. Proportion likely to be suitable for DMT referral highlighted, based on milder cognitive severity and clinical frailty (data only provided for those with recent MMSE and estimated Rockwood). *Where recent MMSE was not available, those with Rockwood <5 were judged to be potentially suitable (n=16). AD, Alzheimer’s disease; DMT, disease-modifying therapy; MMSE, Mini-Mental State Examination.

177 patients in the specialist cognitive service with a diagnosis of AD. Of these, 68 had MMSE ≥20 and Rockwood ≤5; and a further 16 had no recent MMSE, but a Rockwood ≤5 (indicating likely MMSE ≥20), providing 84 potentially suitable patients for DMT (figure 2).

Contraindications to DMTs were then considered based on standard clinical trials exclusion criteria. 4/84 (5%) had a relative contraindication, and 10/84 (12%) had an absolute contraindication to therapy. These included evidence of cerebral amyloid angiopathy, anticoagulant use, MRI intolerance or contraindication, recent substance abuse, current psychosis and territorial infarction. Relative contraindications were also noted in four people as non-aspirin antiplatelet for example, clopidogrel/ticagrelor or active malignancy.

Therefore, once MMSE, clinical frailty and contraindications had been considered, 40% (70/177) of patients with AD were considered potentially eligible, meaning that overall 14% (70/500) of all cases reviewed at the specialist cognitive clinics were potentially eligible for DMTs (figure 2).

## Discussion

By examining clinical case notes from 1017 patients, comprising 517 patients assessed in memory services and 500 patients in a specialist cognitive service, we provide the most substantial estimate of the proportion of patients likely to be referred for DMTs for AD to date. We considered diagnosis, together with cognitive severity, clinical frailty, MRI exclusions and treatment with anticoagulation. This revealed that 32% of patients in memory services are likely to be referred for triaging prior to consideration of DMT for AD; that is, up to 32% of the population seen in memory clinics may require access to amyloid biomarker testing. With an average memory clinic caseload of 815 and 80 nationally accredited memory clinics in England and Northern Ireland, potentially over 20 000 people per year will need access to such confirmatory investigations.^16^

Once access to confirmatory investigations and increased MRI availability is considered, as in specialist cognitive services, this falls to 14% of patients likely to be eligible for DMT. 14% is close to current estimates from US and European settings, where DMTs are beginning to be delivered.^17 18^ However, demographic data in our study suggest that it is likely that the population seen in the cognitive clinic represents only a portion of those we judged as potentially suitable for triage from the memory services, meaning that the proportion of those referred who will ultimately be suitable for DMT is likely to be substantially less than 14%. Systems need to be set up to deal with this potential large mismatch between referral and ultimate eligibility in order to avoid overwhelming services.

A significant issue is that due to the lack of biomarker testing in community memory clinics, the clinical suspicion of AD is likely to be incorrect in at least 30% of cases.^19^ Accurate diagnosis, which requires biomarker support would reduce the number ultimately being eligible for therapies. A secure diagnosis of AD with beta-amyloid accumulation is only possible with fluid biomarkers or PET imaging, which are currently not usually available to memory services within the UK; a clear and urgent area for improvement. Equitable availability will aid accurate diagnosis in community memory services, thereby reducing the number of patients being referred for triage and assessment for therapies for which they would not be suitable and avoid bottlenecks in accessing treatment for suitable patients.

Our work is not without limitations. The retrospective design may have introduced bias based on historical referral patterns. Diagnostic uncertainty was poorly captured, and so we employed an inclusive approach to ensure a lack of exclusion. We also lacked ethnicity data at the specialist cognitive clinic.

Estimated eligibility rates for DMTs vary widely, depending on the patient setting. For example, in an Irish specialist cognitive service, where patients were preselected based on CSF positivity for AD biomarkers, 57% met eligibility criteria, with the most common reason for unsuitability being cognitive score below threshold cut-offs.^20^ In a specialist cognitive clinic in Sweden, with full fluid biomarker and imaging profiling, of 404 consecutive patients, 192 (47.5%) had appropriate diagnoses, and of these, 22% were excluded due to low cognitive scores.^21^ A recent US study, using a population-based cohort, attempted to estimate eligibility according to clinical trial entry criteria.^18^ Patients with mild cognitive impairment or mild dementia were preselected using amyloid-PET, which is not widely available in the UK. Of those with mild cognitive impairment or dementia and positive amyloid-PET, 47.3% were eligible for inclusion into the lecanemab trial and 43.9% into the aducanumab trial. These proportions fell to 8% and 5.1%, respectively, when full exclusion criteria were employed.^18^ However, exclusion criteria used in that study were extensive and would be more limited in real-world use of DMTs.

However, given previous lack of DMTs, those with mild cognitive impairment may not currently seek out referral, leading to underestimates of population eligibility when using current UK memory service data. A recent French study estimated likely eligibility rates from prevalence rates of mild cognitive impairment (MCI) and AD, suggesting 1.65 million people have MCI due to AD; and that 311 043 have amyloid-positive AD with mild-stage cognitive severity. These data are based on prevalence estimates, rather than sampling, and without actual measures of cognitive severity or clinical frailty, are likely to overestimate numbers of potentially eligible patients.

An important recent study estimated real-world demand for DMTs in the UK using an anonymised patient record database from two National Health Service trusts, including records from 82 386 people.^22^ That study similarly applied inclusion criteria from the donanemab and lecanemab trials and considered appropriate diagnosis of AD, MMSE Score equivalent over 20, numbers able to undergo imaging and exclusions based on vascular burden on MRI, and other reasons including bleeding disorders and exclusionary neurological conditions. They also considered numbers likely to agree to treatment, based on take-up of current treatment with cholinesterase inhibitors. They arrived at 464 patients taking up treatment, out of 1432 with appropriate diagnoses, or 32%. This figure agrees with our estimate of 32% of potential referrals for further consideration for treatment and strengthens support for our estimate, given the different populations and methods used in the two studies.

Important and notable differences emerged between patients seen at the contrasting centres we studied. There was a higher rate of diagnosis of ‘mixed’ dementia or non-specific phenotypes at the memory services. This likely reflects lower access to confirmatory diagnostic tests including CSF and amyloid-PET, and lower availability of MRI scanning and access to neuroradiological expertise and multidisciplinary meetings. On the other hand, a higher prevalence of rarer dementias such as frontotemporal dementia was seen in the specialist cognitive service. This is likely due to a combination of referral patterns from other centres, access to diagnostic tests and a research focus on these conditions in that centre, leading to regular follow-up of patients with those conditions. A higher proportion of patients at the specialist cognitive service were under 70 and had diagnoses of subjective cognitive impairment, which also reflects referral patterns to the service being targeted to younger-onset dementias, and in part based on referral criteria to the specialist clinic.

Information on MRI contraindications or risk of haemorrhage (eg, due to anticoagulants or cerebral microbleeds) would need to be assessed during triaging when considering DMTs. *APOE* genotype can also be used to evaluate risk of side effects with DMTs, with risk of ARIA-E increasing from 5.4% for E4 non-carriers, to 10.9% for heterozygotes, to 32.6% for E4 homozygotes for lecanemab.^23^ Exactly how and when *APOE* would be tested is not yet clear, as there are additional implications for family members. However, if patients opt for genetic testing as part of risk evaluation for DMTs, it would further reduce the numbers accessing DMTs.

It is crucial to ensure support mechanisms are put in place for patients who do not meet eligibility criteria, at a minimum to maintain current levels of service and ideally to improve these via evidence-based interventions, in order to ensure that this majority cohort is not inadvertently excluded from services.

Service design to enable equitable access to DMT is currently ongoing; however, ‘known unknowns’ regarding likely demand are a limitation to implementation. While there are limitations on the accuracy of our estimates, given current barriers to early clinical presentations and referral, our study provides predicted numbers based on real-world community cohorts. Ensuring that pathways ensure equity of access, given the varying populations served in memory clinics, remains a challenge. The use of easily deployed, culturally fair screening tools may help with effective and timely triage. It is likely that demand for diagnostic services among those with early cognitive concerns will increase following licensing and NICE technology appraisals, placing further demands on already overstretched services. Local memory services may refer as many patients as feasible with a possible diagnosis of AD, to avoid delays or denying access to DMTs. This means that effective triaging will be essential, based on exclusion criteria from imaging, and confirmatory investigations. It is likely that over time, eligibility criteria will widen, as has occurred with MS DMT and stroke thrombolysis, due to increasing therapeutic options and emerging data supporting widening access criteria. These factors all need to be taken into account when planning services and managing clinician and patient expectations.

### Summary

This service evaluation has shown that approximately 32% of patients seen in memory services are potentially eligible for triaging for DMTs for AD, with numbers reducing further once confirmatory investigations are performed. This information can be used to model capacity for triaging and for delivery of these new treatments for AD.
